# Acupuncture for chronic prostatitis: A systematic review and meta-analysis protocol

**DOI:** 10.1097/MD.0000000000010615

**Published:** 2018-04-27

**Authors:** Tianzhong Peng, Ying Cheng, Yuhao Jin, Na Xu, Taipin Guo

**Affiliations:** a Nanchang Hongdu Hospital of Traditional Chinese Medicine, Nanchang, Jiangxi Province; b Yunnan University of Traditional Chinese Medicine, Kunming, Yunnan Province, China.

**Keywords:** acupuncture, chronic prostatitis, protocol, systematic review

## Abstract

**Background:**

Chronic prostatitis (CP) is a prevalent genitourinary condition. Considering its safety profile, acupuncture can be an option treating CP symptoms. The aim of this review is to undertake a systematic review to estimate the effectiveness and safety of acupuncture on CP.

**Methods:**

We will search all randomized controlled trials for CP in August 2018 in the databases of MEDLINE, Cochrane Library, Web of Science, EMBASE, Springer, WHO International Clinical Trials Registry Platform (ICTRP), China National Knowledge Infrastructure (CNKI), Wan fang, Chinese Biomedical Literature Database (CBM), PsycInfo, Chinese Scientific Journal Database (VIP), and other available resources. Languages are limited as English and Chinese. Search terms used are will “acupuncture,” and “chronic prostatitis,” “non-bacterial prostatitis,” “abacterial prostatitis.” And duplicates will be screened. The primary outcomes consisted of improvement rate and pain relief evaluated by The National Institutes of Health Chronic Prostatitis Symptom Index (NIH-CPSI) index. Secondary outcomes include the recurrence rate and side effects, such as pneumothorax, discomforts, and infection.

**Results:**

This study will demonstrate an evidence-based review of acupuncture for chronic prostatitis.

**Conclusion:**

The study will provide clear evidence to assess the effectiveness and side effects of acupuncture for chronic prostatitis.

**Ethics and dissemination:**

There is no requirement of ethical approval and it will be in print or disseminated by electronic copies.

**PROSPERO registration number:**

CRD42018088834.

## 1 Introduction

### 1.1 Description of the condition

Chronic prostatitis (CP)^[1–3]^ is a prevalent condition and is commonly accompanied by lower urinary tract symptoms. CP is also a complex andrological dysfunction with a lifetime prevalence of 1.8% to 8%^[4]^ for men of all ages and races, most prominent in the elderly. It has ranked fourth among the 20^[5]^ principal diagnostic diseases in the United States. The estimates also suggest that many cases might be under-diagnosed and undertreated by physicians.^[6]^

Most patients with CP have varying degrees and manifestations of voiding symptoms, a deferent duct obstruction, recurrent of urinary tract infections, and ejaculatory pain. Evidence also supports that there is a relationship^[7]^ between male infertility and prostatitis.

The National Institutes of Health (NIH) describes prostatitis into 4 categories.^[8]^ This research will cover randomized controlled trials (RCT) mainly for category III, or chronic prostatitis/chronic pelvic pain syndrome (CP/CPPS), which accounts for 90%^[9]^ cases of CP.

The pathogenesis of category III prostatitis is poorly understood^[10,11]^; yet, substantial evidence suggests it is a multifactorial condition, where interactions exist between an infectious or inflammatory initiator, neurological injury, uric acid level, psychological factors, and dysfunction in the immune system.

General diagnosis is based on the presence of white blood cells, present in expressed prostate secretions (EPS), semen, or urine using a colorimetric assay, standard microbiological method, and culturing.^[12–14]^

Novel therapies^[15,16]^ include alpha-blocker, anti-inflammatory therapy, physiotherapy, neuroleptics, anti-anxiolytics, antidepressants, etc. However, treatment of noninflammatory CP is difficult, and pharmacological therapies proved unsuccessful in most cases. Further, using anti-inflammatory drugs is associated with serious side-effects of gastrointestinal and cardiovascular disorders. So non-pharmacological treatments such as acupuncture^[17–19]^ are therefore increasingly attractive. To date, acupuncture is often used for CP and chronic pain relief in east countries.^[20]^

To evaluate the evidence for the effectiveness of acupuncture in CP, we will undertake a systematic review and meta-analysis of RCTs. Based on the evidence of prospectively designed and RCTs in a well-defined population, we will use a validated outcome (The National Institutes of Health Chronic Prostatitis Symptom Index [NIH-CPSI]), and make treatment recommendations for physicians.

### 1.2 Description of the intervention

According to this approach, acupuncture's effect will depend on specific acupoints, the stimulation intensity, frequency, and repetition. Then, we can provide a basis for defining the adequacy of acupuncture and sham interventions.

### 1.3 How the intervention might work?

Acupuncture, one of the most commonly used non-pharmacological therapies, has been considered a form of sensory nerve stimulation.^[21,22]^ It has been used for relieving pain based on the evidence of biological mechanism. In east countries, acupuncture has been broadly employed for chronic conditions such as myofacial pain, muscle disorders, and neurological disorders.^[23]^

The theories of TCM believe^[24]^ that the human body is a hierarchical and holistic organism, mutually interacting through meridian systems. The root cause of pain is stagnation of meridian system,^[25]^ which will cause full or partial blockade neurovascular flows in regions. Acupuncture can help to regulate and redistribute the neurovascular flow in the body, thereby to improve neuromuscular dysfunction and relieve chronic fibromyalgia.

### 1.4 Why it is important to conduct this review?

We will systematically review and critically appraised the published literatures, and compare acupuncture with various types of control interventions. Different conclusion can be drawn, and the meta-analysis of RCTs will provide sufficient data.

### 1.5 Objectives

The primary goal of this systematic review is to appraise the effectiveness and safety of acupuncture on CP. Based on the evidence, we may recommend an effective therapy.

## 2 Methods

### 2.1 Study registration

PROSPERO registration number is CRD42018088834. This protocol report is structured according to the Preferred Reporting Items for Systematic Reviews and Meta-Analyses Protocols (PRISMA-P) statement guidelines.^[26]^

### 2.2 Inclusion criteria for study selection

#### 2.2.1 Types of study

To evaluate the efficacy of acupuncture on chronic CP, all relevant studies will be retrieved as full reports for detailed evaluation. We will include prospective RCTs of any form of acupuncture. The language will be limited to Chinese and English. The animal mechanism studies, case reports, non-RCTs, or RCT protocol are excluded.

#### 2.2.2 Types of participants

There are no restrictions on population ages. The chronic prostatitis selected is type III with unknown causes. The definitions of chronic prostatitis or chronic prostate disorder are included. Patients with acute medical conditions are excluded.

#### 2.2.3 Types of intervention

The review comprises clinical trials with the treatment of acupuncture. We will study the types of acupuncture including fine needle, floating needle, electro-acupuncture, etc. Any study that did not satisfy the inclusion criteria will be excluded. Studies to compare the effect of acupuncture stimulations methods or acupoints will be excluded. The sham procedure will be assessed to ensure reliable effects.

#### 2.2.4 Types of outcome measures

The main outcome measure consists of NIH-CPSI index, expressed prostatic secretion (EPS) score, quality of life during intervention, and at follow-up. Secondary outcomes include the recurrence rate and side effects, and data on adverse events will be collected through observation by acupuncturists or self-reported by participants.

#### 2.2.5 Data sources

We will search database in MEDLINE, EBASE, Cochrane Library, Springer, CNKI, Wanfang, WHO International Clinical Trials Registry Platform (ICTRP), CBM, and VIP. The languages are Chinese and English.

#### 2.2.6 Search strategy

The following search keyword or combination subject terms are used: RCT (controlled clinical trial); acupuncture (e.g., “acupuncture” or “TCM acupuncture” or “fine needle acupuncture,” and “electro- acupuncture” or “fire needling”; chronic prostatitis; abacterial prostatitis; prostatodynia; prostatalgia; prostate pain). For Chinese databases, these search concepts will be precisely translated. The search strategies for Medline are summarized in Table 1.

**Table 1:**
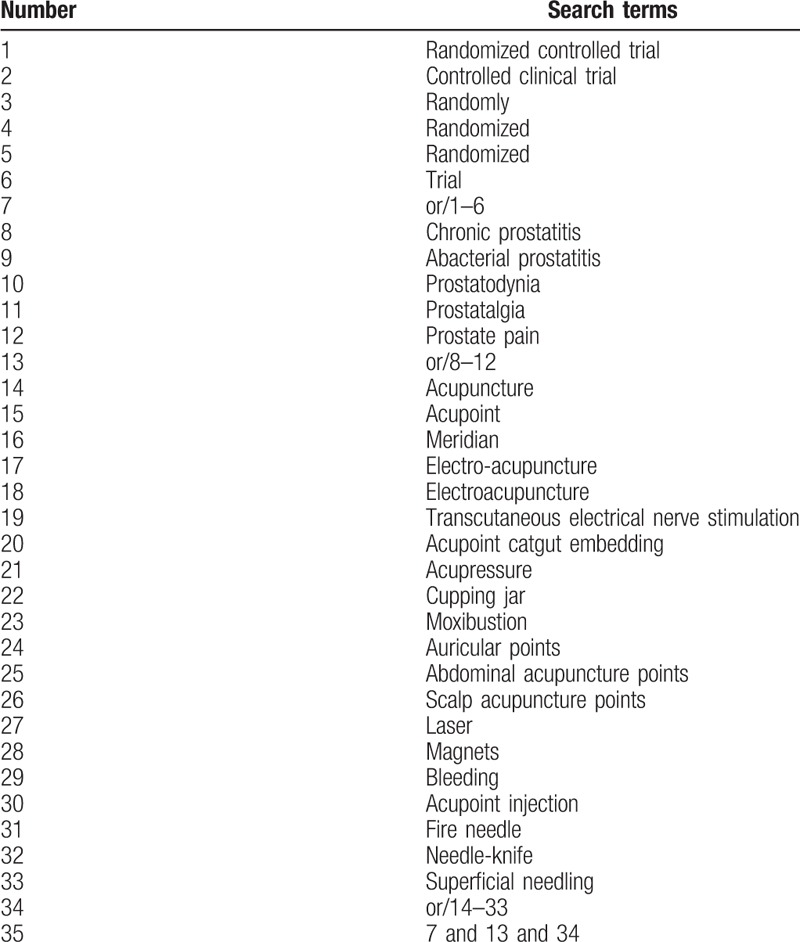
Medline search strategy.

### 2.3 Data collection and analysis

#### 2.3.1 Selection of studies

**Figure 1:**
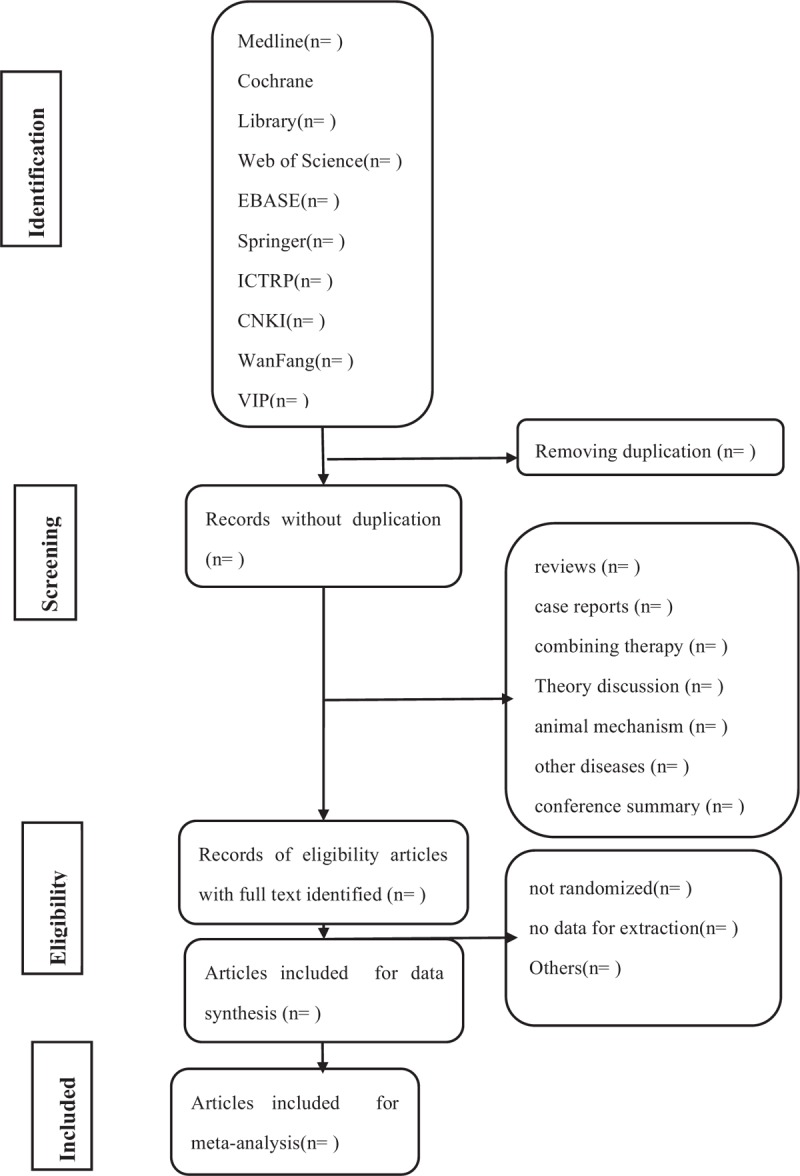
Flow diagram of studies identified.

Selection, data extraction, and quality assessment will be performed independently by 2 reviewers (TZP and YC). All relevant articles of full text are investigated. When the 2 reviewers cannot agree on the selection process through consultations, the third reviewer (TPG) will ultimately make the decision. Translations of the full Chinese reports will be undertaken by YHJ. The primary selection process is shown in a PRISMA flow chart (Fig. 1)

#### 2.3.2 Data extraction and management

Data will be extracted systematically by predefined, standardized methods on study design, participant characteristics, results, and statistical information. Evaluation score will be calculated using multiple criteria, including description of randomization, blinding, and withdrawals.

#### 2.3.3 Assessment of risk of bias and reporting of study quality

Cochrane risk of bias tool will be used to score RCTs and assess for bias with the Cochrane Collaboration's risk-of-bias assessment method and complete the Standards for Reporting Interventions in Controlled Trials of Acupuncture (STRICTA) checklist. Jadad Scale will be used to evaluate for methodological quality.

For unclear data, the author is contacted. And any study that does not satisfy the inclusion criteria will be excluded. The final decisions will be made by the third author (TPG).

#### 2.3.4 Measures of treatment effect

We will use mean differences (MDs) with 95% confidence intervals (95% CIs) to analyze continuous data. Other forms of data will be changed into MD values. Risk ratio and random-effects models are used for significant heterogeneity.

#### 2.3.5 Unit of analysis issues

Separate multiple meta-analyses for each treatment objective will be used. Multiple control groups are included, and pooled analyses of the control groups are used.

#### 2.3.6 Management of missing data

For missing or incomplete data, we will try to contact the authors. If the data are still incomplete, we will screen it out.

#### 2.3.7 Assessment of heterogeneity

Clinical heterogeneity will be explored through data synthesis, to identify any variations, Review Manager (vesion 5.3,Copenhagen, The Nordic Cochrane Centre, The Cochrane Collaboration, 2014)^[27]^ and forest plot are used to illustrate the relative strength of curative effect. If significant heterogeneity is detected, secondary analyses based on variations in RCT characteristics will be used.

#### 2.3.8 Assessment of reporting biases

If >10 trials are included, we will use funnel plots to evaluate reporting biases.

#### 2.3.9 Data synthesis

The mean change in each of the outcome measures will be compared. Weighted mean differences (WMD) and a random-effects model with 95% CI will be calculated using Review Manager V5.3.^[27]^

#### 2.3.10 Subgroup analysis

We will perform a subgroup analysis according to sham procedure and different outcomes.

#### 2.3.11 Sensitivity analysis

A sensitivity analysis will be conducted according to predefined criteria.

## 3 Discussion

CP begins with some form of inductive factor within the prostate gland^[28]^:infection, injury, dysfunctional voiding, allergy, etc., which may cause inflammation or neurogenic damage in and around the prostate. If the treatment is insufficient, peripheral and central sensitization will happen.

It has been found recently that an altered autonomic nervous system response^[29]^ in men with CP/CPPS. Since no reliable data or experiences to substantiate a traditional treatment, in patients who have developed a chronic condition, physiotherapy of acupuncture may provide benefit.

A report published^[30]^ by WHO Consultation on Acupuncture states that acupuncture has been practiced on a wide range of physiological disorders including pain, infection, neurological disorders, urogenital disorders, etc. However, most of the mechanisms have not been able to explain how acupuncture might work.

This review will comprise RCTs with patients from different countries in either Chinese or English. The study will provide the first evidence that acupuncture may be considered an effective treatment for CP and how it is shown to be superior to pharmaceutical therapies in relieving symptoms and improving male reproductive function. Meanwhile, it will evaluate the secondary analyses to explore reasons for potential heterogeneity.

## Author contributions

**Data curation:** Yuhao Jin.

**Investigation:** Na Xu.

**Methodology:** Na Xu.

**Software:** Yuhao Jin.

**Supervision:** Tainpin Guo.

**Writing – original draft:** Tianzhong Peng, Ying Cheng.

**Writing – review and editing:** Tainpin Guo.
